# Neuritin Inhibits Notch Signaling through Interacted with Neuralized to Promote the Neurite Growth

**DOI:** 10.3389/fnmol.2017.00179

**Published:** 2017-06-08

**Authors:** Pan Zhang, Xing Luo, Zheng Guo, Anying Xiong, Hongchang Dong, Qiao Zhang, Chunyan Liu, Jingling Zhu, Haiyan Wang, Na Yu, Jinli Zhang, Yu Hong, Lei Yang, Jin Huang

**Affiliations:** ^1^The Key Laboratory of Xinjiang Endemic and Ethnic Diseases, Department of Biochemistry, Shihezi University School of MedicineShihezi, China; ^2^School of Medicine, Hangzhou Normal UniversityHangzhou, China

**Keywords:** neuritin, neuralized, notch signaling, neurite growth, neural development, nerve plasticity

## Abstract

Neuritin plays a key role in neural development and regeneration by promoting neurite outgrowth and synapse maturation. However, the mechanism of neuritin in modulating neurite growth has not been elucidated. Here, using yeast two-hybrid we screened and discovered the interaction of neuritin and neuralized (NEURL1), which is an important regulator that can activate Notch signaling through promoting endocytosis of Notch ligand. And then we identified the interaction of neuritin and neuralized by co-immunoprecipitation (IP) assays, and clarified that neuritin and NEURL1 were co-localized on the cell membrane of SH-SY5Y cells. Moreover, neuritin significantly suppressed Notch ligand Jagged1 (JAG1) endocytosis promoted by NEURL1, and then inhibited the activation of Notch receptor Notch intracellular domain (NICD) and decreased the expression of downstream gene hairy and enhancer of split-1 (HES1). Importantly, the effect of neuritin on inhibiting Notch signaling was rescued by NEURL1, which indicated that neuritin is an upstream and negative regulator of NEURL1 to inhibit Notch signaling through interaction with NEURL1. Notably, recombinant neuritin restored the retraction of neurites caused by activation of Notch, and neurite growth stimulated by neuritin was partially blocked by NEURL1. These findings establish neuritin as an upstream and negative regulator of NEURL1 that inhibits Notch signaling to promote neurite growth. This mechanism connects neuritin with Notch signaling, and provides a valuable foundation for further investigation of neuritin’s role in neurodevelopment and neural plasticity.

## Introduction

Neuritin (also known as CPG15) was identified in a screen for activity-regulated genes involved in synaptic plasticity (Nedivi et al., 1993) and encodes an extracellular signaling molecule (Naeve et al., 1997). Further studies revealed that neuritin supplies are abundant and activity-independent at early developmental stages when neuritin promotes dendritic and axonal growth and synaptic maturation (Nedivi et al., 1998; Cantallops et al., 2000; Javaherian and Cline, 2005). In addition, neuritin facilitated the survival of cultured cortical neurons obtained during the embryonic developmental period by preventing apoptosis (Putz et al., 2005). However, as maturation progresses neuritin expression declines, and its supplies become activity-dependent and limited to a handful of plasticity zones (auditory and visual areas; Corriveau et al., 1999). Knockout of neuritin in mice delayed developmental maturation of axonal and dendritic arbors and formation of mature synapses, with many dendritic spines that initially lacked functional synaptic contacts (Fujino et al., 2011). Furthermore, neuritin is also involved in the re-establishment of neuronal networks during nerve regeneration and repair, and functions in learning and memory (Fargo et al., 2008; Karamoysoyli et al., 2008; Zhao et al., 2015). Recombinant neuritin promoted structural and functional recovery of sciatic nerve injury (Wang et al., 2016), inhibited nerve cell apoptosis and accelerated neurite regeneration and recovery of motor function after spinal cord injury in rats (Gao et al., 2016). In addition, Neuritin protein is an important effector of nerve growth factor and androgen in enhancing peripheral nerve regeneration following injury (Fargo et al., 2008; Karamoysoyli et al., 2008). These results indicate that neuritin plays a key role in neural development and regeneration by promoting neurite outgrowth and synapse maturation. However, as a downstream effector of neurite outgrowth (Nedivi et al., 1998), the molecular mechanism explaining how neuritin promotes neurite growth remains obscure.

Notch signaling is an evolutionarily conserved cell-cell signaling mechanism involved in cell fate decisions during different cellular and developmental processes (Artavanis-Tsakonas et al., 1999; Bray, 2006), including neural development. It is found that Notch-ligand interactions among neighboring neurons mutually restrict neurite growth and affect final neuron size. Upregulation of Notch activity inhibited axon extension and caused neurite retraction in neurons with extending neurites (Berezovska et al., 1999; Sestan et al., 1999). Forced expression of the Notch intracellular domain (NICD) or downstream effector hairy and enhancer of split-1 (HES1) significantly inhibited axon outgrowth in the rostral hindbrain, diencephalon and spinal cord (Shi et al., 2011). Furthermore, antagonizing Notch signaling promoted neurite extension (Sestan et al., 1999; Shi et al., 2011), and blocking Notch activation improved regeneration after axonal injury (El Bejjani and Hammarlund, 2012). These results suggest that changes in Notch activity contribute to differences in neuronal capacity to grow and differentiate.

Here, we report that neuritin interacts with NEURL1 and show that neuritin inhibits Notch signaling through NEURL1 to promote neurite growth. This mechanism connects neuritin with Notch signaling and explains their joint participation in nervous system development.

## Materials and Methods

### Two-Hybrid Library Screening

The open reading frame region of the human neuritin gene was amplified with PCR and was cloned into pGBK-T7 (Clontech) to generate the bait plasmid pGBK-T7-neuritin. A human fetal brain cDNA library (Clontech) was screened with the pGBKT7-neuritin bait plasmid according to the manufacturer’s instructions. All of the positive clones were repeatedly screened on the synthetic nutrition drop-out culture medium plates. After initial clustering on positive colonies with PCR and electrophoresis, identified prey cDNA-encoding proteins were sequenced and the sequence alignment was analyzed.

### Expression Plasmids

The open reading frame region of the human neuritin gene was amplified by PCR and subcloned into the pcDNA3.1 expression vector (Invitrogen), generating the plasmid pcDNA3.1-neuritin with a 6-histidine (His)-fusion at the carboxyl terminus. The expression vector encoding hemagglutinin (HA)-tagged full length Jagged1 (JAG1) or Flag-tagged full length NEURL1 in pcDNA3.1 was provided by Addgene. The sequences of all constructs were verified by DNA sequencing.

### Knockdown of Gene Expression Using siRNA

A neuritin siRNA plasmid was generated by using the pBS/U6 vector (Wan et al., 2005). Briefly, a 22 nucleotide (nt) oligo (oligo 1) at nt 333–351, 406–424 or 456–474 of the human neuritin coding region was first inserted into the pBS/U6 vector after digesting the vector with ApaI (Promega) and HindIII (Promega). The inverted motif containing a 6-nt spacer and five thymidines (oligo 2) was then subcloned between the Hind III and EcoR I (Promega) sites of the intermediate plasmid to generate pBS/U6-Neuritin-siRNA-1, -2 and -3. siRNA plasmids were transfected into cells as described below, and the efficiency of knockdown was verified by western blotting.

### Cell Culture and Treatment

To clarify the molecular mechanisms underlying neuritin’s effects, 293T cells were used, while SH-SY5Y cells and PC12 cells were used to observe the neurite outgrowth. Human SH-SY5Y neuroblastoma cells and 293T cells were routinely cultured in DMEM containing 4.5 g/L glucose and 4 mM L-glutamine (HyClone) supplemented with 10% (v/v) fetal bovine serum (Gibco). PC12 cells were maintained in RPMI 1640 plus 10% horse serum and 5% fetal bovine serum. Cells were maintained at 37°C in a humidified atmosphere of 95% air/5% CO_2_ until 70%–80% confluence was attained. SH-SY5Y cells were transfected by electroporation using Lonza nucleofection technique as previously described (Maess et al., 2011). The 293T cells and PC12 cells were transfected using Lipofectamine 2000 (Invitrogen) according to the manufacturer’s protocol. Based on the protocols developed as previously described (Ahmed et al., 2015), cells were subject to cell number and neurite growth analyses. Recombinant neuritin (hNRN1)-containing (500 ng/mL, His treatment as control) medium was added on day 0 and then replaced with fresh hNRN1-containing media every 2 days for a total of 2 days (SH-SY5Y cells) or 7 days (PC12 cells). Cell number and neurite length were determined using ImageJ software with the NeuronJ plug-in.

### Immunoprecipitation and Western Blotting Analysis

Cells were lysed in buffer containing 150 mM NaCl, 1% Triton-X 100, 0.5% sodium deoxycholate, 1 M Tris-HCl, pH 7.5 and 1% phenylmethylsulfonyl fluoride (Solarbio) on ice for 30 min. For immunoprecipitation (IP) analysis, lysates were incubated with agarose beads (GE Healthcare) and different antibodies as specified in the figures. Immunocomplexes were washed with washing buffer (150 mM NaCl, 1% Triton-X 100, 0.5% sodium deoxycholate, 1 M Tris-HCl, pH 7.5) and then eluted from the beads with elution buffer (150 mM NaCl, 1% Triton-X 100, 0.5% sodium deoxycholate, 0.1% SDS, 1 M Tris-HCl, pH 7.5). All samples were detected by western blotting. For western blotting analysis, lysates were centrifuged at 15,616× *g* for 10 min at 4°C, and protein concentration in the supernatant was determined by Super-Bradford Protein Assay Kit (CWBIO) and normalized. After electrophoretic separation by SDS-PAGE, proteins were transferred to polyvinylidene difluoride membranes (Millipore). All blots were performed as described previously (Huang et al., 2007) with indicated antibody and visualized using Immobilon Western Chemiluminescent HRP Substrate (Millipore). Antibodies were obtained from the following sources: anti-His, anti-Flag and anti-HA, Sigma; anti-neuritin, anti- NEURL1 and anti-NICD, Abcam; anti-HES1, Cell Signaling Technologies; anti- actin, ZSGB-BIO.

### Immunofluorescence

Cells were seeded on cover slips and incubated at 37°C until 50%–60% confluence was attained. Cells were fixed in 4% paraformaldehyde for 30 min at 4°C, and then blocked in 10% (w/v) normal goat serum in PBS for 30 min at room temperature. Cells were incubated for 2 h with indicated antibodies (antibodies against Neuritin, NEURL1, caveolin and microtubule-associated protein2 (MAP2) were from Abcam), followed by goat anti-mouse FITC-conjugated IgG (sigma) or Rhodamine-conjugated AffiniPure Goat Anti-mouse IgG (ZSGB-BIO). Digital pictures were taken with a Zeiss LSM 510 Meta Laser Scanning Confocal Microscope or an Olympus IX70 Inverted Research Microscope. Adobe Photoshop CS6 and Illustrator CS6 were used to prepare the figures.

### Analysis of Neurite Outgrowth

Phase contrast digital micrographs of cell monolayers were collected using the Carl Zeiss Axio Vert.A1 (Carl Zeiss) and analyzed using ImageJ software with the NeuronJ plug-in. A neurite is defined as a cellular projection as long or wide as the cell soma. The length of neurites was measured along the axis of each cell’s longest neurite. Five fields per experiment were analyzed, with 20 or more cells measured per field. Differences in the number of cells with neurites between cell populations were analyzed using Fisher’s exact test (SPSS17.0). Data for the length of neurites were not normally distributed, so the Mann-Whitney U non-parametric test was used instead.

## Results

### The Proteins Interact with Neuritin were Screened

**Figure 1:**
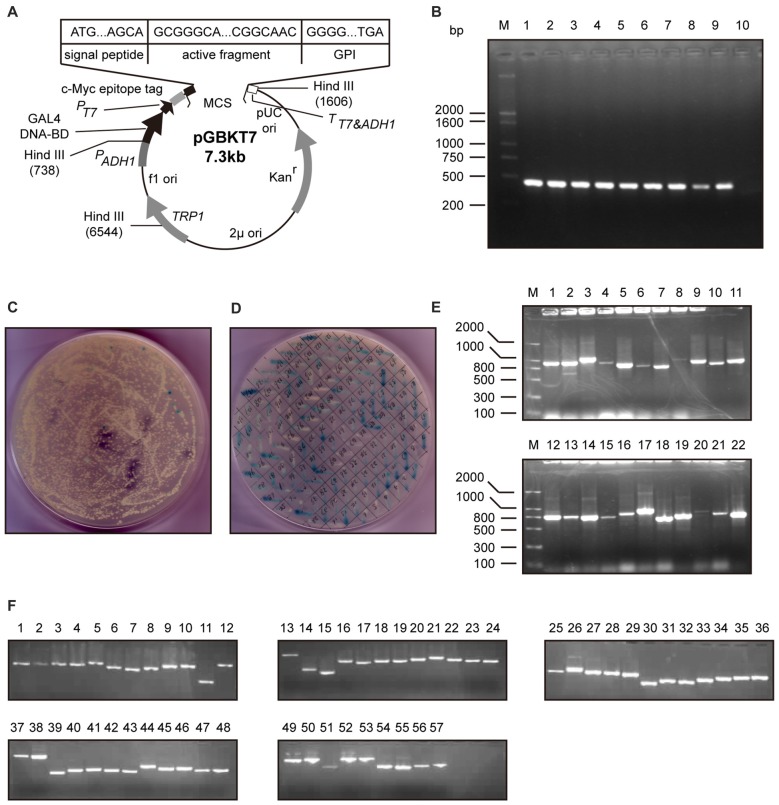
Screening of proteins interacting with neuritin. **(A,B)** Construction of the bait vector pGBKT7-neuritin in the yeast two-hybrid system. The open reading frame region of the human neuritin gene was amplified by PCR and subcloned into the pGBKT7 vector. The full-length neuritin coding sequence of 426 bp was inserted between EcoRI and BamH1. **(C,D)** Positive clones were chosen after repeated screening on SD/-Trp/-Leu/-Ade/-His/+3-AT nutrition drop-out culture medium plates contains X-α-Gal, then amplified. pGBKT7-neuritin was transformed into the yeast two-hybrid reporter strain AH109 and colonies over 2 mm diameter were chosen. **(E)** Initial clustering of positive colonies was performed with PCR and electrophoresis. There were no electrophoretic bands in some lanes and multiple bands in other lanes. **(F)** The last clustering of positive clones through electrophoresis identified a total of 57 prey cDNA-encoded proteins and sequenced.

To explore the proteins interacting with neuritin, we performed yeast two-hybrid screening. The entire neuritin protein was fused in-frame with the GAL4 DNA-binding domain as the bait (Figures 1A,B), and a human fetal brain cDNA library was screened (Figures 1C,D). DNA sequence analysis identified 57 positive clones (Figures 1E,F), one of which was NEURL1 (Table 1), encoding a human membrane-associated E3 ligase (Lai et al., 2001).

**Table 1:** Candidate genes coding proteins for Neuritin binding.

Genbank accession number	Gene symbol	Gene name	Category
AF029729.1	Neuralized	Neuralized mRNA	Nerve related
NM_032796.2	SYAP1	Synapse associated protein 1	Nerve related
BC002654.1	TUBB6	Tubulin, beta 6	Cytoskeleton related
BC009512.2	TUBA1B	Tubulin, alpha 1b	Cytoskeleton related
NM_006407.2	JWA	Cytoskeleton related vitamin A responsive protein	Cytoskeleton related
NM_001614.2	ACTG1	Actin, gamma 1	Cytoskeleton related
BC018641.2	EEF1A1	Eukaryotic translation elongation factor 1 alpha 1	Translation related
BC048105.1	EIF4A2	Eukaryotic translation initiation factor 4A	Translation related
NM_000985.2	RPL17	Ribosomal protein L17	Ribosome related
NM_000988.2	RPL27	Ribosomal protein L27	Ribosome related

*Clustering positive clones were sequenced and sequence alignments were analyzed. The table shows the 10 most highly aligned genes, including the gene Neuralized*.

### Neuritin Interacted with NEURL1 and Reduced NEURL1 Expression

**Figure 2:**
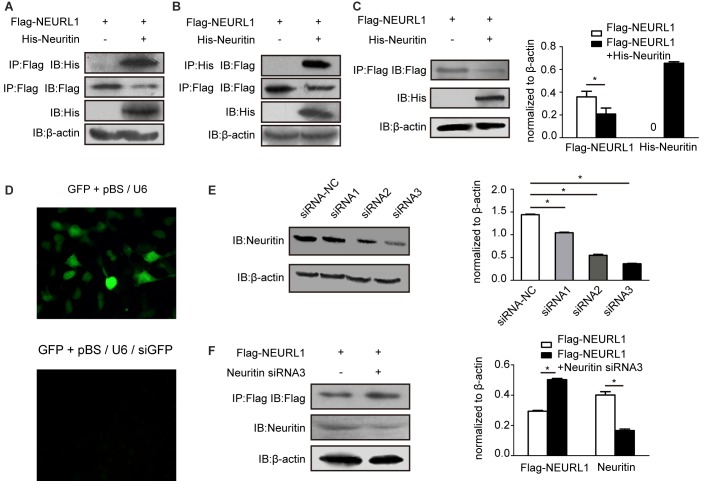
Neuritin interacts with NEURL1 to reduce NEURL1 expression. **(A,B)** Exogenous interaction of neuritin and NEURL1 was detected by immunoprecipitation (IP) in 293T cells. His-neuritin and Flag-NEURL1 were overexpressed in 293T cells; pcDNA3.1 empty vector was used as a control. Specific antibody against Flag was used to immunoprecipitate exogenous NEURL1 proteins from total cell lysates, and the immunocomplex was detected by western blotting using anti-His antibody. The expression levels of His-neuritin and Flag-NEURL1 in cells were detected as indicated in the lower panels. Reverse co-IP was performed in the same way. **(C)** Overexpression of neuritin decreased NEURL1 expression levels. Flag-tagged NEURL1 was co-transfected with either His-tagged neuritin or empty vector in 293T cells. At 48 h after transfection, cell lysates were prepared and subjected to SDS-PAGE and immunoblot analysis using anti-Flag, anti-His or anti-β-actin antibodies individually. *p < 0.01, 2-sided Fisher’s exact test. **(D)** pBS/U6 plasmid knockdown efficiency in 293T cells. Cells were transfected with GFP, pBS/U6 or pBS/U6/siGFP and showed significant knockdown of GFP expression by pBS/U6/siGFP compared to pBS/U6. **(E)** Knockdown of neuritin expression in 293T cells transfected with neuritin siRNA-NC or neuritin siRNAs. Expression levels of neuritin and β-actin were measured by western blotting analysis and densitometry. *p < 0.01, 2-sided Fisher’s exact test. **(F)** Neuritin siRNA3 elevated NEURL1 protein levels in 293T cells transfected with neuritin siRNA3. Levels of neuritin, NEURL1 and β-actin were measured by western blotting analysis and densitometry. *p < 0.01, 2-sided Fisher’s exact test.

To determine the interaction of neuritin and NEURL1 in mammalian cells, we performed co-IP experiments using total cell lysates prepared from human 293T cells overexpressing His-Neuritin and Flag-NEURL1. Western blotting analysis of the immunoprecipitates with anti-His antibody revealed the presence of neuritin in the anti-Flag immunoprecipitates (Figure 2A). In the reciprocal experiments, His antibody was observed to co-immunoprecipitate NEURL1 (Figure 2B).

We then examined whether the interaction of neuritin and NEURL1 affects NEURL1 levels in cells. The plasmid expressing neuritin was co-transfected with Flag-tagged NEURL1 into 293T cells, and the results of western blotting demonstrated that the expression level of NEURL1 was reduced by neuritin compared with the empty vector transfection control (Figure 2C). To confirm the effect of neuritin on NEURL1, we employed an RNA interference approach to silence neuritin and tested the inhibition efficiency (Figure 2D). And neuritin siRNA-3 demonstrated strongerinhibition of neuritin expression than the other siRNAs (Figure 2E) and was subsequently used to test the effect of neuritin suppression. Consistently, western blotting measurements showed that neuritin depletion was associated with increased NEURL1 (Figure 2F), while neuritin overexpression led to reduced NEURL1, further supporting the notion that neuritin represses NEURL1 levels.

### Neuritin Suppressed NEURL1-Mediated JAG1 Endocytosis and Inhibited Notch Signaling Activation

**Figure 3:**
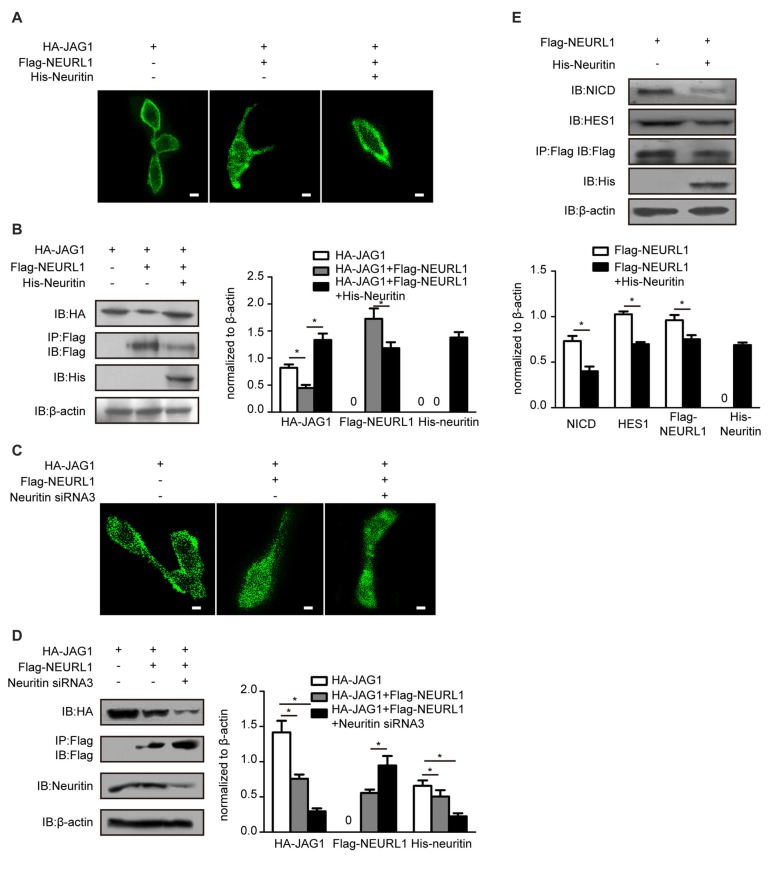
Neuritin suppresses NEURL1-mediated JAG1 endocytosis and Notch signaling. **(A,B)** Neuritin overexpression suppressed NEURL1-mediated JAG1 endocytosis and degradation in 293T cells expressing HA-JAG1. Cells were transfected with Flag-NEURL1 only or with Flag-NEURL1 and His-neuritin. JAG1 endocytosis was visualized by immunofluorescence. Corresponding experiments show expression levels of HA-JAG1 and other cellular proteins by western blotting analysis and densitometry. *p < 0.01, 2-sided Fisher’s exact test. Scale bar represents 10 μm. **(C,D)** Knockdown of neuritin increased NEURL1-mediated JAG1 endocytosis and degradation in 293T cells expressing HA-JAG1 and Flag-NEURL1 transfected with or without neuritin siRNA-3. JAG1 endocytosis was visualized by immunofluorescence. Protein expression in transfected cells was detected by western blotting and densitometry. *p < 0.01, 2-sided Fisher’s exact test. Scale bar represents 10 μm. **(E)** Expression of Flag-NEURL1 in 293T cells transfected with or without His-neuritin. The NICD and HES1 were detected by western blotting analysis and densitometry. pcDNA3.1 empty vector was used as a control. *p < 0.01, 2-sided Fisher’s exact test.

NEURL1 as an E3 ligase promotes endocytosis of Notch ligands to activate Notch signaling (Pavlopoulos et al., 2001). We hypothesized that neuritin was involved in the endocytosis of notch ligand JAG1. Immunofluorescence showed that endocytosis of JAG1 promoted by NEURL1 was dramatically decreased by overexpression of neuritin in 293T cells (Figure 3A), whereas immunoblotting showed that the JAG1 level was increased with the reduction in endocytosis in neuritin-transfected 293T cells (Figure 3B). Conversely, neuritin siRNA-transfected cells exhibited an obvious increase in JAG1 endocytosis and decreasing JAG1 levels (Figures 3C,D).

**Figure 4:**
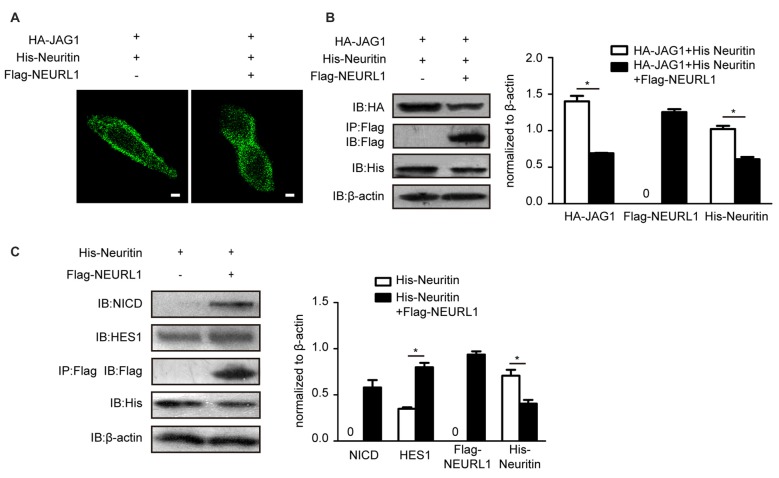
Overexpressing NEURL1 rescues the inhibition of Notch signaling by neuritin. **(A,B)** NEURL1 overexpression rescued the suppression of JAG1 endocytosis and degradation by neuritin in 293T cells expressing HA-JAG1 and transfected with His-neuritin only or with His-neuritin and Flag-NEURL1. JAG1 endocytosis was visualized by immunofluorescence. Corresponding experiments show expression levels of HA-JAG1 and other cellular proteins by western blotting analysis and densitometry. *p < 0.01, 2-sided Fisher’s exact test. Scale bar represents 10 μm. **(C)** Inhibition of Notch signaling by neuritin was rescued by NEURL1 in 293T cells expressing His-neuritin and transfected with or without Flag-NEURL1. The NICD and HES1 were detected by western blotting and densitometry. pcDNA3.1 empty vector was used as a control. *p < 0.01, 2-sided Fisher’s exact test.

The endocytosis of JAG1 is indispensable for activating Notch signaling (Le Borgne et al., 2005). To test whether neuritin-reduced JAG1 endocytosis affects Notch activation, we examined levels of the NICD, which is the activated Notch receptor. Western blotting results showed that the NICD was significantly reduced in NEURL1 transfected cells with neuritin compared to that without neuritin (Figure 3E). To examine whether HES1, the downstream target of Notch signaling, was also affected, western blotting was used to detect the expression level of HES1 in neuritin-overexpressing cells (Figure 3E). Results showed that HES1 expression was partially decreased in Neuritin and NEURL1 co-transfected cells compared with NEURL1 transfected cells (Figure 3E). Moreover, neuritin-suppressed endocytosis of JAG1 was mediated by NEURL1 (Figures 4A,B), since ectopic expression of NEURL1 was able to restore the inhibitory effects of neuritin on the NICD and HES1 (Figure 4C), supporting the notion that the impact of neuritin on Notch signaling is mediated by NEURL1.

### Neuritin Co-Localized and Interacted with Endogenous NEURL1 in SH-SY5Y Cells

**Figure 5:**
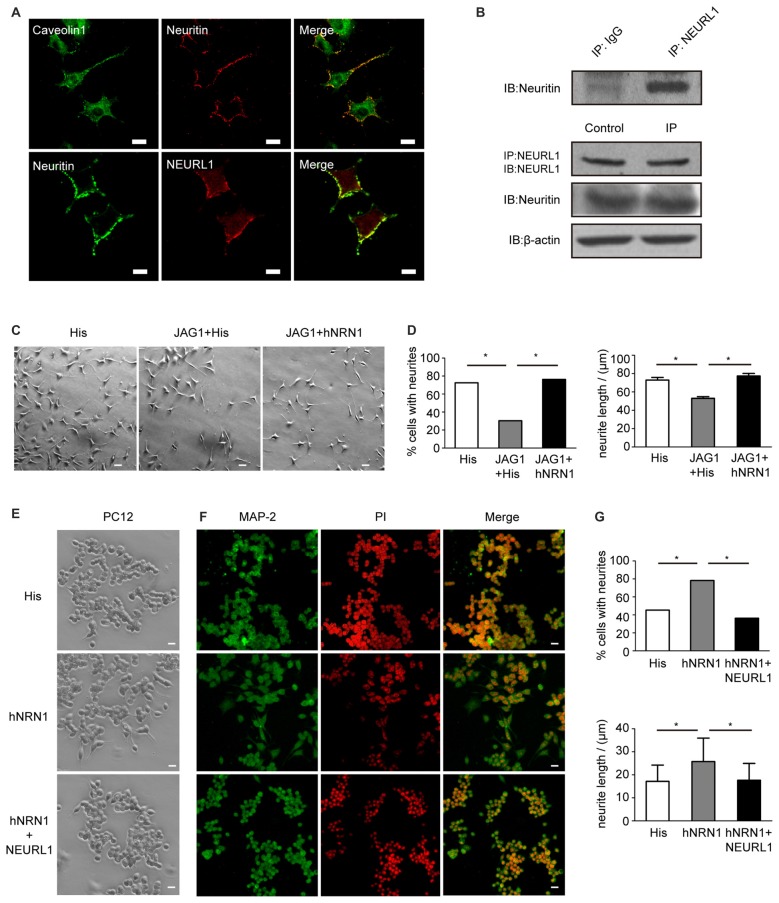
Neuritin interacts with NEURL1 and restores the retraction of neurites caused by Notch activation. **(A)** Colocalization of neuritin and NEURL1 in SH-SY5Y cells was analyzed by immunofluorescence microscopy. Photomicrographs show an overlap between neuritin-positive signals and NEURL1, and also with caveolin, a marker of lipid rafts. Scale bar represents 20 μm. **(B)** Endogenous interaction of neuritin and NEURL1 was detected by IP using IgG as a control. **(C)** SH-SY5Y cells transfected with pcDNA3.1-HA-JAG1 or pcDNA3.1 empty vector as a control were treated with hNRN1 (500 ng/mL). His peptide was used as a negative control. Representative images of neurites are shown in the designated conditions. Scale bar represents 50 μm. **(D)** The percentage of cells from **(C)** recorded as having one or more neurites in each group (*p < 0.01, 2-sided Fisher’s exact test). The summary histogram shows the mean length of neurites analyzed by Mann-Whitney U test (*p < 0.01). **(E)** PC12 cells treated with hNRN1 (500 ng/mL; His peptide as negative control) were transfected with pcDNA3.1-Flag-NEURL1 or pcDNA3.1 empty vector as a control. Representative images of neurites are shown in the designated conditions. Scale bar represents 50 μm. **(F)** Photomicrographs of PC12 cells immunostained with anti-microtubule-associated protein2 (MAP2) antibody; cell nuclei were counterstained with PI. Scale bar represents 50 μm. **(G)** The percentage of cells from **(C)** recorded as having one or more neurites in each group (*p < 0.01, 2-sided Fisher’s exact test). The histogram shows the mean length of neurites analyzed by Mann-Whitney U test (*p < 0.01).

To assess the association of neuritin with NEURL1 in neural cells, immunofluorescence assays were performed to determine the subcellular localization of neuritin and NEURL1 in SH-SY5Y neuroblastoma cells. Neuritin was localized to the cell membrane coincided with expression of NEURL1 and caveolin, a marker of lipid rafts (Figure 5A). Co-IP was employed to confirm the interaction of endogenous neuritin and NEURL1. In agreement with the above experiments, neuritin was also present in anti-NEURL1 immunoprecipitates (Figure 5B). The results confirmed that neuritin indeed interacts with NEURL1 in neurons.

### Neuritin Restored the Notch-Induced Neurites Retraction

To determine if the inhibition of Notch signaling by neuritin was associated with neurite growth, SH-SY5Y cell populations were evaluated for the growth of neurites that represent a key feature of neurons. The cell phenotype observed after 2 days of neuritin treatment showed that a large proportion of the SH-SY5Y cells displayed multiple neurites, whereas these processes appeared less abundant in HA-JAG1 transfected cells (Figure 5C). To provide an objective measurement of neurite outgrowth, over 100 cells were measured in each cell population to determine the length and number of cell projections. Referencing the length of the neurites against the cell body, both the number and length of neurites appeared to be substantially increased in SH-SY5Y cells treated with hNRN1. Statistical analysis confirmed that the number of cells with neurites has increased from 30.38% in HA-JAG1-transfected control cells to 76.3% in hNRN1-treated cells (*p < 0.01, Figure 5D). Similarly, neurite lengths in HA-JAG1-transfected cells were confirmed to be significantly shorter than in neuritin-treated cultures (*p < 0.01, Figure 5C). These results indicate that hNRN1 rescued the Jag1-induced neurites retraction in SH-SY5Y cells.

To investigate whether NEURL1 reversed the effect of neuritin on neurite growth, we observed that undifferentiated PC12 cells (without neurite) treated with hNRN1 displayed neurite growth, but this effect was blocked by NEURL1 (Figure 5E). MAP2 immunostaining confirmed this result (Figure 5F). Statistical analyses showed that both the number and length of neurites in NEURL1-transfected PC12 cells were significantly reduced compared to control-transfected cells after hNRN1 treatment (*p < 0.01, Figure 5G). These data thus indicate that the effect of neuritin on neurite growth was mediated by NEURL1.

## Discussion

Studies show that neuritin participates in neural development and nerve regeneration by promoting neurite outgrowth and synapse formation. However, the mechanisms underlying this effect have not been elucidated. Our study revealed that neuritin is an upstream and negative regulator of NEURL1, and inhibits NEURL1-mediated Notch signaling to promote neurite growth.

Using neuritin as bait, we screened a rat embryo brain cDNA pool using the yeast two-hybrid system and obtained 57 genes encoding proteins which may interact with neuritin. Following clustering analysis of the above genes, we observed that NEURL1, which mediates signaling in the Notch pathway, shared many similar physiological functions with neuritin during neural development. Therefore, we focused on the interaction between neuritin and NEURL1. We determined that neuritin interacts with NEURL1 through forward and reverse Co-IP in 293T cells, and verified that neuritin suppressed levels of NEURL1. NEURL1 acts as a regulator by promoting endocytosis of ligands to activate Notch signaling (Lai et al., 2001; Pavlopoulos et al., 2001). In particular, endocytosis of Delta/JAG1 is an important indicator of NEURL1 activation of Notch signaling (Deblandre et al., 2001; Delwig et al., 2006; Koutelou et al., 2008; Hansson et al., 2010). Our research showed that neuritin significantly suppressed JAG1 endocytosis promoted by NEURL1; meanwhile, western blot results showed higher levels of JAG1 in cells, suggesting less degradation of JAG1. Furthermore, we observed the influence of neuritin on Notch receptor and downstream effectors. Significantly, overexpressing neuritin reduced the generation of the NICD, the activated Notch receptor, and decreased HES1, the downstream target gene of Notch signaling. Our experiments also demonstrated that NEURL1 mediated the influence of neuritin on Notch signaling, since overexpression of NEURL1 not only reversed the inhibition of neuritin on JAG1 endocytosis but also rescued the reduction of NICD and HES1 caused by neuritin. Thus, we can draw the preliminary conclusion that neuritin is an upstream and negative regulator of NEURL1 and inhibits Notch signaling through interaction with NEURL1.

**Figure 6:**
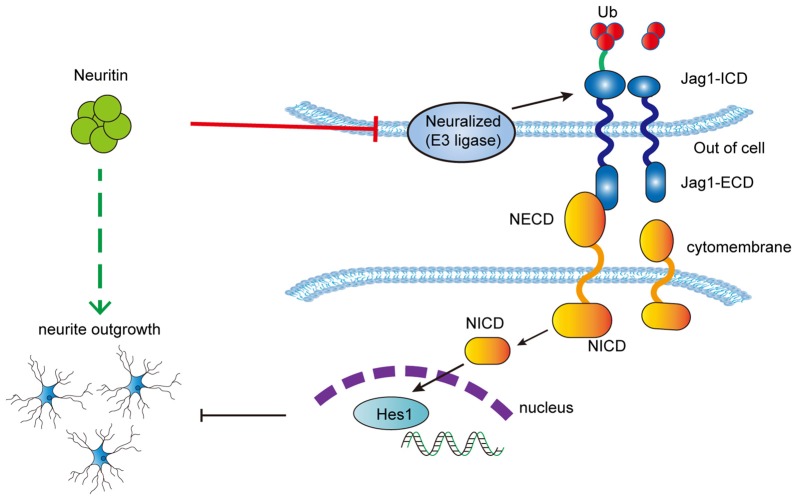
Schematic depicting the proposed mechanism of neuritin inhibition of the Notch pathway to promote neurite growth.

To explore whether the inhibitory effect of neuritin on the Notch pathway contributed to the promotion of neurite growth, neuroblastoma SY5Y cells with neural characteristics were used to observe neuritin function on neurite growth. It was first determined that neuritin co-localized with NEURL1 in the cell membrane and interacted with endogenous NEURL1 in SY5Y cells. Importantly, recombinant neuritin treatment restored the retraction of neurites caused by Jag1. To determine if NEURL1 could rescue the effect of neuritin on neurite growth, we found that neuritin-stimulated neurite growth in undifferentiated PC12 cells without neurites was partially blocked by NEURL1, which indicated that NEURL1 could reverse the stimulatory effect of neuritin on neurite growth. Taken together, we conclude that neuritin inhibits Notch signaling through NEURL to promote neurite growth (Figure 6).

Interestingly, there is a shift between neuritin and Notch signaling during the neural development; as one aspect wanes, the other increases. At an early developmental stage, activity-independent high expression of neuritin causes rapid growth of nerve cell neurites and synapse formation (Nedivi et al., 1998; Cantallops et al., 2000; Javaherian and Cline, 2005), while Notch signaling decreases (Hartl et al., 2008). After maturation, when neuritin is in decline (Lee and Nedivi, 2002), the formation of neuronal contacts results in activation of Notch receptors, leading to restriction of neuronal growth and a subsequent arrest in maturity (Sestan et al., 1999). However, there is currently no literature to connect these events together. To our knowledge we provide the first piece of evidence illustrating the interaction between neuritin and Notch. This may explain the high expression of neuritin during early neurological development, when inhibition of Notch signaling leads to rapid neurite outgrowth. The later decline in neuritin expression during maturation removes or relieves inhibition of Notch signaling, resulting in the cessation of neurite growth and retraction of neurites. We therefore propose that neuritin and Notch may co-regulate the balance between neurite growth and retraction to maintain the structure and size of the CNS during neurodevelopment. While these results still need to be confirmed *in vivo*, our findings on the mechanism of neuritin will provide a valuable foundation for further investigation of the role of neuritin in neurodevelopment and neural plasticity.

## Author Contributions

JH, LY, PZ and XL: conception and design; ZG, XL, PZ, AX, HD and QZ: performing the experiments; CL, NY and HD: development of methodology; PZ, HW, JZ (11th author) and YH: analysis and interpretation of data; JH, PZ, XL, JZ (8th author), HW and ZG: writing, review and/or revision of manuscript; JH and LY: administrative, technical or material support.

## Conflict of Interest Statement

The authors declare that the research was conducted in the absence of any commercial or financial relationships that could be construed as a potential conflict of interest.
